# An online survey on public awareness of drug clinical trials in inland cities of northern China

**DOI:** 10.3389/fpubh.2024.1276536

**Published:** 2024-04-11

**Authors:** Li Wang, Ruiguang Zhang, Jingxuan Liu, Rui Xu, Lili Zhao, Enze Li, Yurong Zhang

**Affiliations:** ^1^Department of Scientific Research, Faculty of General Family Medicine, the First Affiliated Hospital of Xi’an Medical University, Xi’an, Shaanxi, China; ^2^Department of Health Statistics, Faculty of Public Health, Shaanxi University of Chinese Medicine, Xi’an, Shannxi, China; ^3^Xi’an Evidence Based Pharmaceutical Technology Co., Ltd, Xi’an, China

**Keywords:** drug clinical trials, public, awareness of drug clinical trials, willingness to participate the drug clinical trials, in northern China

## Abstract

**Objective:**

The aims of this survey were to investigate the public awareness of drug clinical trials (DCTs) and willingness to participate the DCTs, and provide references for propaganda and science popularization of DCTs.

**Methods:**

A self-designed questionnaire named “an online survey questionnaire on public awareness of DCTs” was used to conduct an online survey from January to March 2022. The demographic characteristics and the response of participants to the awareness and willingness to participate the DCTs were collected. The factors affecting the public awareness of DCTs were analyzed by single factor and binary logistic regression analysis.

**Results:**

One thousand three hundred eighty valid questionnaires were collected, and the respondents’ awareness rate of DCTs was 61.1%. Thirteen demographic characteristics including age, gender, education, occupation, work fields, household type, marital status, city type, income, medical insurance, medical expenditure, pressure to seek medical care, financial pressure, both significantly affected the qualified rate of participants’ awareness of DCTs (*p* < 0.001) by single factor analysis. Binary logistic regression analysis indicated that education level, work fields, city type, medical insurance, and medical expenditure affected independently the participants’ awareness rate of DCTs (*p* < 0.001). 52.9% of the participants were willing to take part in DCTs. “to promote medical progress” (54.4%) or “believe doctors” (31.1%) were the most frequent reasons for subjects participating in DCTs.

**Conclusion:**

The public awareness rate of DCTs and the willingness to participate in drug clinical were significantly affected by the demographic characteristics of subjects. Thus, targeting the needs of the public, propaganda, and science popularization of DCTs should be carried out and served public health.

## 1 Introduction

With the advancement of the economy, living standards, and national cultural in China, there has been a discernible shift towards a more scientifically informed public awareness regarding clinical trials. Consequently, an increasing number of pharmaceutical enterprises are directing significant attention towards harnessing clinical research resources within China. Presently, China has emerged as a pivotal hub for clinical trials, owing to its vast population scale, abundant medical infrastructure, and comparatively lower research expenditure (1). Encumbered by the high costs associated with new drug research and limited clinical resources elsewhere, foreign pharmaceutical enterprises are progressively relocating their research centers to China.

The 14th Five-Year Plan has introduced a range of policies, notably including “Opinions on Reforming the Review and Approval System of Drugs and Medical Devices”,^1^ aimed at overhauling the regulatory framework governing drugs and medical devices while fostering biomedical innovation. Consequently, the research and development capabilities pertaining to innovative drugs, vaccines, and high-end medical devices have experienced consistent augmentation. This has led to a notable increase in the number of new drugs and medical devices advancing into clinical stages. Consequently, the demand for clinical trial resources, such as subjects, has witnessed a substantial annual upsurge. The quantity of registered clinical trials in China approached nearly 10,000 (2), yielding an impressive output value of 700 billion US dollars from 2016 to 2020. According to data from Informa Pharma Intelligence, the average annual growth rate for Phase I to Phase IV clinical trials stood at 20% (3). These data underscore the heightened investment in funds for new drug research and development and signify the escalating health consciousness within China.

According to statistics, approximately 500,000 individuals participate to the DCTs in China annually, this figure that remains relatively small compared to the nation’s vast population base. However, the emergence of the COVID-19 pandemic (4) has reignited public interest in keywords such as “drug/vaccine clinical trials,” “clinical trials,” and “subjects.” Clinical trials serve as crucial avenues for offering additional treatment options, particularly for patients lacking access to effective remedies (5, 6), thereby presenting an opportunity for disease management and potential cure. Nonetheless, obstacles to subject participation in clinical trials consistently result in recruitment failure (7). Hence, it is crucial to explore public awareness of DCTs for subject recruitment. Unfortunately, a thorough investigation into the public awareness of DCTs in China has yet to be undertaken. Thus, the present study aims to fill this gap by investigating public awareness of DCTs and identifying key factors influencing this awareness through the design and implementation of a questionnaire. The findings seek to furnish a theoretical foundation for enhancing public engagement in clinical trials.

## 2 Methods

### 2.1 Study population

This investigation constitutes a cross-sectional survey which employed non-random convenience and snowball sampling methodologies. From January to March 2022, healthy adults aged 18 and above, possessing autonomy, adept in independent completion of electronic questionnaires, and exhibiting high cooperative tendencies, were recruited for participation. It is important to note that all participants voluntarily enrolled in this study. Excluding individuals with mental disorders, cognitive impairment, and inability to independently complete all items in the survey questionnaire.

### 2.2 Recruitment and enrollment

Adhering to the principles delineated by Professor Yan Yan in “Medical Statistics” (8), the sample size was determined to be 5–10 times the count of independent variables, hence yielding a calculated sample volume of 420 individuals. To address potential errors and bolster questionnaire effectiveness, the sample size was augmented by 10%, culminating in a confirmed cohort of 470 individuals.

The survey questionnaire was formulated and exported in the form of a two-dimensional code from the “Jinshuju” online data platform. Before the formal survey commenced, 20 preliminary questionnaires were disseminated, revealing incomplete responses. Measures were implemented to constrain each IP address to a single response and to mandate the completion of all items, reinforcing the scientificity and preciseness of the survey. Healthy individuals over 18 years of age from five provinces in the northern region of China were chosen as the study cohort. A liaison officer was designated to each province to disseminate information online within the community, including the objective and significance of survey, instructions for completing the questionnaire and notes on filling out the questionnaires. WeChat groups were established in each community for the distribution and retrieval of questionnaires. These questionnaires were scanned and completed using the two-dimensional code, enabling online data automatic collection.

Participants were tasked with anonymously addressing 42 survey inquiries, with response validity hinging on completeness and the absence of missing elements. Subsequent to an elucidation of the research objectives, questionnaire completion protocols, and principles of confidentiality, the electronic questionnaire was shared with respondents following the procurement of their informed consent. Respondents were encouraged to independently progress through the questionnaire, receiving standardized instructions and annotations for item completion. The formulation of the questionnaire deliberately avoided suggestive prompts. Data in Excel format was exported from “Jinshuju” data platform and underwent reconciliation and cleaning by the researcher, including logical error checking to enhance the accuracy of source material, ultimately facilitating the identification of valid surveys while excluding those with incomplete or insincere responses. A total of 1,462 surveys were collected in, with 82 deemed invalid, resulting in an effective response rate of 94.4%.

### 2.3 Development and validation of the survey questionnaire

This research initiative assembled a team of 5 investigators dedicated to questionnaire development, comprising 2 individuals with senior professional designations and 3 with intermediate professional affiliations. Their primary duties encompassed literature review and analysis, determination of questionnaire items, data collection and statistical analysis, questionnaire refinement, assessment of questionnaire reliability and validity, and ultimately, questionnaire finalization.

Employing English search terms such as “drug/vaccine clinical trials,” “clinical trials,” “subjects,” “cognitively,” “willingness & drug clinical trials,” “attitude & drug clinical trials” and “awareness,” comprehensive literature searches were conducted across prominent databases including PubMed, Embase, The Cochrane Library, Biomedical Literature Databases, CNKI, and Wanfang Data Knowledge Service Platform. The exploration encompassed subject heading terms from the inception of the databases until December 2021. Following the removal of duplicate literature, the remaining titles, abstracts, and full texts underwent sequential review. Encompassed study types included clinical practice guidelines, systematic reviews, clinical randomized controlled trials, cohort studies, observational studies, expert opinions, and other research. Exclusion criteria comprised duplicated publications or translated literature, incomplete data, and inaccessible full texts. Drawing from the outcomes of the literature review and analysis, the metrics of the questionnaire were defined, and the questionnaire items were initially formulated.

Following collaborative discussion, the initial iteration of the Online Survey Questionnaire on Public Awareness of DCTs was formulated, encompassing two sections. The first section covers general demographic information, consisting of 13 entries such as age, gender, education, occupation, work fields, household type, marital status, city type, income, medical insurance, medical expenditure, pressure to seek medical care, and financial pressure. The second section entails the public awareness survey of DCTs, incorporating two dimensions: public awareness of DCTs and willingness to participate the DCTs. Within the Public Awareness Survey of DCTs, 20 items were related to cognitive assessment of DCTs, encompassing aspects such as new drug clinical trial procedures, allocation methods, compensation for damages to subjects, free trial drugs and related physical examinations, ethics and more. Each item was evaluated on a 3-point scale: “yes,” “no,” “do not know” correlating to respective scores of 2, 1, and 0. A total of 20 items need to score according to the participants’ options of clinical trial-related questions in the questionnaires. Participants who achieved a score exceeding 24 points were deemed to exhibit a proficient understanding of DCTs. Each item on the scale was positively evaluated, with higher aggregate scores indicative of an elevated comprehension of DCTs. Moreover, the Public Willingness Survey to Participate the DCTs comprised 9 items addressing the inclination to partake in clinical trials, factors motivating participation, and related considerations. The outcome variables focused on public awareness of DCTs.

### 2.4 Assessment of the Questionnaire’s reliability and validity

Reliability was assessed through the utilization of Cronbach’s alpha coefficient, while validity underwent evaluation via Bartlett’s sphericity test. A significance level of *p* < 0.05 was stipulated for statistical significance. Generally, a Cronbach’s α coefficient exceeding 0.700 is deemed acceptable (9), with a value falling within the range of 0.800 to 0.900 indicating an outstanding level of internal consistency reliability (10), thus meeting the benchmark for a foundational research instrument. The questionnaire was subjected to reliability and validity testing using SPSS 26.0, resulting in a total Cronbach’s α coefficient of 0.973 and a KMO coefficient of 0.975, both approximating 1. Additionally, the Bartlett’s sphericity test produced an approximate *χ*^2^ value of 28933.950, with a *p* < 0.001, aligning with the intended objectives. These outcomes signify robust reliability and validity within the questionnaire, rendering it suitable for implementation.

### 2.5 Quality control

The survey questionnaire is distributed online, and all respondents are willing to fill it out voluntarily. Measures are taken to ensure that all items in the questionnaire are completed before submission. Therefore, the collected questionnaires are those with all items completed. Through repeated distribution and snowball like online forwarding, extensive and multiple online education sessions have been conducted, expanding the scope of dissemination and increasing the number of participants. Through these methods, participant compliance is ensured, participant acceptance is improved, and sample size requirements are also met. Additionally, it is essential to select appropriate measurement tools and assessment methodologies, establish uniform standards and guidelines for researchers, and conduct thorough data review and verification to enhance data quality.

### 2.6 Statistical analysis

Utilizing SPSS 26.0 for data analysis, categorical data description entails the use of frequency and constituent ratio, Measures obeying normal distribution were analyzed by t-test and F-test for hypothesis testing of their overall means, whereas ($\bar{x}$ *± s*) is utilized for describing continuous data. While measures not obeying normal distribution were analyzed by non-parametric tests. The *χ*^2^ test is applied to compare discrepancies in the awareness of DCTs across demographics. Logistic regression analysis is employed to gauge the influence of demographic variables on public awareness of DCTs. Noteworthy demographic variables are singled out through univariate analysis and integrated into the ultimate logistic regression model. For the variables encompassed in the regression model, their odds ratios (OR) and 95% confidence intervals are computed. A significance threshold of *p* < 0.05 denotes statistical significance.

## 3 Results

### 3.1 Demographic characteristics statistics

A total of 1,462 questionnaires were collected in the survey, among them, 82 questionnaires with incomplete information or obvious filling errors were removed and 1,380 valid questionnaires (94.4%) were used to analyze the study.

This study comprised 1,380 valid questionnaires, with 1,380 distributed across 5 provinces and municipalities in northern China. Specifically, there were 328 from Shaanxi, 345 from Shanxi, 259 from Gansu, 247 from Ningxia Hui Autonomous Region, and 201 from Henan Province. The survey respondents from Shaanxi and Shanxi were almost occupied 50%, all with a relatively even distribution of the population in each province and a good representation of the sample.

The study included 562 male participants (40.7%) and 818 female participants (59.3%). Individuals under the age of 40 constituted 81.23% of the total respondents. Additionally, 47.9% of the participants had attained a bachelor’s degree or higher level of education. Among the participants, the most prevalent occupations were students (43.5%), followed by individuals employed in public institutions (19.6%), and those working in enterprises (12.7%). Regarding household registration type, 56.6% of participants hailed from urban areas. Approximately 43.8% of the participants were married, and 47.3% reported a monthly income level ranging from 0 to 2000 CNY. In terms of medical insurance, the majority of participants were enrolled in medical insurance for urban and rural residents (36.8%), followed by urban employee medical insurance (31.5%), and self-funded medical insurance (7.7%). Furthermore, 38.2% of respondents stated that their medical expenditure accounted for less than half of their monthly income. Notably, over half of the participants experienced varying degrees of medical (56.4%) and financial pressure (67.9%). The demographic characteristics are summarized in Table 1.

**Table 1:** Demographic characteristics statistics.

Item	Class	*n*	%
Age	18–20 years old	484	35.07
	21–30 years old	336	24.35
	31–40 years old	301	21.81
	41-60 years old	225	16.30
	> 60 years old	34	2.46
Gender	Male	562	40.72
	Female	818	59.28
Education	Under junior college	481	34.86
	Junior college	239	17.32
	Undergraduate	470	34.06
	Postgraduate	190	13.77
Occupation	Administration organization	38	2.75
	Public institution	271	19.64
	Enterprise	175	12.68
	Student	600	43.48
	Farmer	99	7.17
	Retired	31	2.25
	Other	166	12.03
Work fields	Non-medical related	737	53.41
	Medical-related	643	46.59
Household type	Urban	599	43.41
	Rural	781	56.59
Marital status	Unmarried	775	56.16
	Married	605	43.84
City type	First-tier city	300	21.74
	Second-tier city	323	23.41
	Third-tier city	246	17.83
	Fourth-tier city and below	511	37.03
Income	0 ~ 2000 CNY	653	47.32
	2001 ~ 3,000 CNY	111	8.04
	3,001 ~ 5,000 CNY	232	16.81
	5,001 ~ 8,000 CNY	227	16.45
	> 8,000 CNY	157	11.38
Medical insurance	Public medical care	124	8.99
	Urban employee medical insurance	435	31.52
	Urban and rural residents medical insurance	508	36.81
	Out-of-pocket medical expenses	106	7.68
	Private/commercial insurance	20	1.45
	Other	187	13.55
Medical expenditure	Negligible	793	57.46
	< 50% of monthly income	527	38.19
	≥50% of monthly income	60	4.35
Pressure to seek medical care	None	602	43.62
	Mild	475	34.42
	Moderate	220	15.94
	Severe	83	6.01
Financial pressure	None	443	32.10
	Mild	459	33.26
	Moderate	317	22.97
	Severe	161	11.67

### 3.2 Awareness rate of DCTs related knowledge for participants

To gauge public awareness of DCTs, we computed the qualified awareness rate among participants (refer to Table 2). Out of 1,380 participants, the qualified awareness rate regarding DCTs stood at 61.1%. Notably, 70.6% of participants affirmed that they had heard of DCTs, while 18.6% stated they were aware but not familiar with them. It is evident that individuals employed in medical-related fields exhibited a notably higher awareness rate compared to those in non-medical related occupations. Awareness rates exhibited variability across different demographic characteristics, spanning from 47.4 to 70.6%. Notably, awareness concerning compensation for injury events arising during clinical trials was notably low, registering at 47.4 and 48.9%, respectively. Additionally, only 59.1% of participants demonstrated an awareness of the basic definition of DCTs.

**Table 2:** Factors affecting the qualified rate of awareness of DCTs.

Item	Class	Unqualified		Qualified		*χ*^2^ test	
		*n*	%	*n*	%	*χ* ^2^	*p*
Age	18–20 years old	267	55.2%	217	44.8%	112.338^***^	0.000
	21–30 years old	69	20.5%	267	79.5%		
	31–40 years old	99	32.9%	202	67.1%		
	41-60 years old	73	32.4%	152	67.6%		
	> 60 years old	17	50.0%	17	50.0%		
Gender	Male	250	44.5%	312	55.5%	16.685^***^	0.000
	Female	275	33.6%	543	66.4%		
Education	Under junior college	325	67.6%	156	32.4%	305.686^***^	0.000
	Junior college	85	35.6%	154	64.4%		
	Undergraduate	98	20.9%	372	79.1%		
	Postgraduate	17	8.9%	173	91.1%		
Occupation	Administration organization	8	21.1%	30	78.9%	201.261^***^	0.000
	Public institution	41	15.1%	230	84.9%		
	Enterprise	37	21.1%	138	78.9%		
	Student	289	48.2%	311	51.8%		
	Farmer	68	68.7%	31	31.3%		
	Retired	21	67.7%	10	32.3%		
	Other	61	36.7%	105	63.3%		
Work fields	Non-medical related	420	57.0%	317	43.0%	240.839^***^	0.000
	Medical-related	105	16.3%	538	83.7%		
Household type	Urban	148	24.7%	451	75.3%	79.857^***^	0.000
	Rural	377	48.3%	404	51.7%		
Marital status	Unmarried	328	42.3%	447	57.7%	13.733^***^	0.000
	Married	197	32.6%	408	67.4%		
City type	First-tier city	94	31.3%	206	68.7%	103.177^***^	0.000
	Second-tier city	61	18.9%	262	81.1%		
	Third-tier city	101	41.1%	145	58.9%		
	Fourth-tier city and below	269	52.6%	242	47.4%		
Income	0 ~ 2000 CNY	323	49.5%	330	50.5%	130.555^***^	0.000
	2001 ~ 3,000 CNY	59	53.2%	52	46.8%		
	3,001 ~ 5,000 CNY	82	35.3%	150	64.7%		
	5,001 ~ 8,000 CNY	38	16.7%	189	83.3%		
	> 8,000 CNY	23	14.6%	134	85.4%		
Medical insurance	Public medical care	42	36.9%	82	63.1%	116.329^***^	0.000
	Urban employee medical insurance	82	18.9%	353	81.1%		
	Urban and rural residents medical insurance	239	47.0%	269	53.0%		
	Out-of-pocket medical expenses	48	45.3%	58	54.7%		
	Private/commercial insurance	8	40.0%	12	60.0%		
	Other	106	56.7%	81	43.3%		
Medical expenditure	Negligible	308	38.8%	485	61.2%	22.782^***^	0.000
	< 50% of monthly income	178	33.8%	349	66.2%		
	≥50% of monthly income	39	65.0%	21	35.0%		
Pressure to seek medical care	None	243	40.4%	359	59.6%	23.537^***^	0.000
	Mild	146	30.7%	329	69.3%		
	Moderate	90	40.9%	130	59.1%		
	Severe	46	55.4%	37	44.6%		
Financial pressure	None	195	44.0%	248	56.0%	25.782^**^	0.000
	Mild	141	30.7%	318	69.3%		
	Moderate	111	35.0%	206	65.0%		
	Severe	78	48.4%	83	51.6%		

### 3.3 The influence of demographic characteristics on the qualified rate of awareness about DCTs by a single factor analysis

To explore the factors influencing the qualified awareness rate of DCTs, we conducted a single-factor analysis to compare differences across various demographic categories. The findings revealed that demographic characteristics such as gender, age, education, occupation, Work fields, household type, marital status, city type, income, medical insurance, medical expenditure, pressure to seek medical care, and financial pressure all significantly impacted the qualified awareness rate of participants, regarding DCTs (Table 2, *p* < 0.001).

### 3.4 Binary logistic regression analysis of influencing the participants’ awareness of DCTs

To mitigate the influence of interactions between factors on participants’ awareness rate of DCTs, binary logistic regression analysis was employed to evaluate the independent effects of each demographic characteristic. In this analysis, demographic characteristics served as independent variables, while qualification status (qualified = 1, unqualified = 0) was the dependent variable. As presented in Table 3, education, field of work, city type, medical insurance, and medical expenditure were identified as independent factors significantly impacting participants’ awareness rate of DCTs (*p* < 0.05).

**Table 3:** Factors influenced the participants’ awareness rate of DCTs by logistic regression analysis.

Variables	Exp (95% CI)	*p*
*Age*		
18–20 years old	1.000	
21 ~ 30 years old	0.587 (0.208 ~ 1.660)	0.315
31–40 years old	0.933 (0.339 ~ 2.565)	0.893
41–60 years old	0.590 (0.230 ~ 1.517)	0.274
> 60 years old	0.994 (0.396 ~ 2.497)	0.991
*Gender*		
Male	1.000	
Female	1.100 (0.835 ~ 1.451)	0.497
*Education*		
Under junior college	1.000	
Junior college	1.719 (1.130 ~ 2.616)	0.011
Undergraduate	2.759 (1.792 ~ 4.247)	0.0011
Postgraduate	4.905 (2.405 ~ 10.002)	0.001
*Occupation*		
Administration organization	1.000	
Public institution	1.323 (0.314 ~ 1.057)	0.577
Enterprise	1.119 (0.314 ~ 1.057)	0.701
Student	1.552 (0.314 ~ 1.057)	0.127
Farmer	0.542 (0.314 ~ 1.057)	0.067
Retired	0.550 (0.314 ~ 1.057)	0.083
Others	0.400 (0.314 ~ 1.057)	0.080
*Work fields*		
Non-medical related	1.000	
Medical-related	3.217 (2.287 ~ 4.527)	0.000
*Household type*		
Urban	1.000	
Rural	1.002 (0.711 ~ 1.410)	0.993
*Marital status*		
Unmarried	1.000	
Married	0.801 (0.477 ~ 1.343)	0.400
*City type*		
First-tier city	1.000	
Second-tier city	0.612 (0.404 ~ 0.928)	0.021
Third-tier city	0.1.140 (0.745 ~ 1.746)	0.545
Fourth-tier city and below	1.015 (0.704 ~ 1.465)	0.936
*Income*		
0 ~ 2000 CNY	1.000	
2001 ~ 3,000 CNY	0.829 (0.394 ~ 1.741)	0.620
3,001 ~ 5,000 CNY	0.613 (0.288 ~ 1.306)	0.205
5,001 ~ 8,000 CNY	0.528 (0.278 ~ 1.003)	0.051
> 8,000 CNY	1.061 (0.546 ~ 2.062)	0.862
*Medical insurance*		
Public medical care	1.000	
Urban employee medical insurance	1.687 (0.948 ~ 3.003)	0.076
Urban and rural residents medical insurance	1.826 (1.077 ~ 3.093)	0.025
Out-of-pocket medical expenses	1.382 (0.927 ~ 2.060)	1.112
Private/commercial insurance	1.505 (0.859 ~ 2.636)	1.153
Other	0.971 (0.329 ~ 2.867)	0.958
*Medical expenditure*		
None	1.000	
< 50% of monthly income	1.758 (0.890 ~ 3.472)	1.104
≥50% of monthly income	2.008 (1.019 ~ 3.956)	0.044
Pressure to seek medical care		
None	1.000	
Mild	1.358 (0.684 ~ 2.805)	0.366
Moderate	1.414 (0.583 ~ 2.235)	0.700
Severe	0.815 (0.413 ~ 1.608)	0.555
*Financial pressure*		
None	1.000	
Mild	0.980 (0.555 ~ 1.732)	0.946
Moderate	1.113 (0.658 ~ 1.881)	0.690
Severe	1.185 (0.708 ~ 1.983)	0.518
Constants	2.033	0.364

Further analysis revealed significant associations between specific demographic characteristics and the awareness rate of DCTs. Specifically, higher education levels were linked to elevated awareness rate. For instance, participants with postgraduate degree exhibited a 4.905-folds increase in awareness rate compared to those with under junior college education (95% CI: 2.405 ~ 10.002). Moreover, individuals engaged in medical-related occupations demonstrated a substantially higher awareness rate, with a 3.217-folds increase compared to those in non-medical related fields (95% CI: 2.287–4.527). City type also exerted a notable influence on participants’ awareness of DCTs. Notably, individuals residing in second-tier cities exhibited a mere 0.612-fold awareness rate compared to those in first-tier cities (95% CI: 0.404–0.928). Additionally, participants covered by medical insurance for urban and rural residents demonstrated a 1.8268-folds increase in awareness rate compared to the general population covered by public medical insurance (95%CI:1.077 ~ 3.093). Furthermore, heightened medical expenditures were associated with enhanced awareness rate of DCTs. Specifically, participants allocating more than 50% of their monthly income to medical expenditure exhibited a 2.008-folds increase in awareness rate compared to those with no medical expenditures (95% CI: 1.019–3.956).

### 3.5 Analysis of willingness to participate in DCTs

In the survey, facilitators and barriers for participants considering participation in DCTs were analyzed. Among the participants, 52.9% expressed willingness to partake in such trials. The primary motivations cited included a desire to “promote medical progress” (54.4%) or a belief in the guidance of doctors (31.1%). Conversely, 47.1% of participants declined participation in DCTs. The foremost reasons for refusal included concerns regarding the risks associated with the trial (62.1%), lack of awareness regarding DCTs (54.6%), reluctance to be treated akin to laboratory mice (32.8%), and unwillingness to allocate additional time and effort (24.3%).

## 4 Discussion

Clinical trials play a crucial role in evaluating the efficacy and safety of new drugs prior to their introduction to the market. The success of clinical trials hinges largely on the recruitment of an adequate number of subjects (11). However, several obstacles hinder the subject recruitment process, including patient preferences, concerns stemming from uncertainty, and apprehensions regarding information and consent procedures, among others (12). Hence, it is imperative to assess the public’s awareness of clinical trials and their willingness to participate in such endeavors. This survey scrutinized the public’s awareness of DCTs, their propensity for participation, and the factors influencing awareness in China.

A comprehensive questionnaire comprising 42 inquiries was developed to elicit respondents’ demographic characteristics, awareness of DCTs, and willingness to participate in such trials in China. Thirteen demographic characteristics were evaluated, including age, gender, education, occupation, field of work, household type, marital status, city type, income, medical insurance, medical expenditure, pressure to seek medical care, and financial pressure. This extensive range of demographic factors surpasses those reported in previous studies (13, 14). This approach allowed for the meticulous capture of a broad range of factors influencing public awareness of DCTs, providing deeper insight into participant attitudes and perceptions.

The 1,380 respondents were widely distributed across several cities in five northern Chinese provinces, with a significant concentration in Shaanxi and Shanxi, comprising 328 and 345 individuals, respectively. The population exhibited a more even distribution across provinces, thus offering a representative sample. In terms of age distribution, participants were evenly spread across four age brackets spanning 18 to 60 years, including 484 individuals aged 18–20, 336 aged 21–30, 301 aged 31–40, and 225 aged 41–60. There were fewer individuals above the age of 60, possibly attributed to the online nature of the survey and the extensive 42-item questionnaire, which may have deterred older adult participation. Challenges in engaging with new media platforms (e.g., WeChat and TikTok) and smartphone technology may have contributed to decreased participation among the elderly due to lower usage frequency. Additionally, students, being more interested in novel experiences such as DCTs, exhibiting relatively high compliance and ease in completing questionnaires, represented a significant proportion of the survey at 43.5%.

The findings revealed that 61.1% of subjects exhibited a qualified awareness rate of drug clinical trials-related knowledge in China. This percentage closely aligns with a survey conducted in Saudi Arabia, where 58% of cancer patients demonstrated awareness of clinical trials, indicating a similar level of awareness between the two studies (15). Moreover, only 3.37% of subjects were familiar with the rights and interests afforded to participants in DCTs, while 58% were knowledgeable about ethical committees and their functions in such trials. Although 70.6% of participants had heard of DCTs, a mere 16.2% had actually participated in them to a limited extent. Additionally, 69.2% of participants were aware of the importance of protecting the privacy and personal information during DCTs, underscoring the significance attached to privacy by the public. However, awareness regarding subjects’ rights, such as the entitlement to receive clinical trial drugs free of charge and the ability to withdraw from the trial at any time for any reason, was relatively low at 57.5 and 50.5%, respectively. This suggests a limited understanding among the public regarding their rights to participate in DCTs and the evolving trend toward patient-centered DCTs.

In recent years, there has been a notable increase in the approval and implementation of DCTs in China (16), leading to a gradual shift in focus toward China in international multi-center trials. The examination of factors influencing awareness rates regarding DCTs in this study encompassed both univariate and binary logistic regression analyses. Univariate analysis revealed that all demographic characteristics collected significantly impacted participants’ awareness rates of DCTs. However, binary logistic regression analysis highlighted that only education level, field of work, city type, medical insurance, and medical expenditure exerted significant effects on participants’ awareness rate of DCTs. These findings suggest that education level, field of work, city type, medical insurance, and medical expenditure are the primary factors influencing public awareness of DCTs in China. This aligns with findings by Primo et al., who identified income and education level as factors related to awareness of cancer clinical trials (17). Similarly, a survey conducted in Korea identified age, religion, financial level, and education level as significant determinants of public awareness of cancer clinical trials (18). These collective results underscore the crucial role of education level as an influential factor in shaping public awareness of DCTs on a global scale. Individuals with higher education levels are likely to have greater exposure to media, internet resources, and other forms of learning, facilitating a deeper understanding of DCTs and related knowledge.

In our survey, 52.9% of participants expressed a willingness to partake in clinical trials, while 50.6% stated that they would recommend their relatives and friends to participate as well. However, this relatively low motivation to engage in clinical trials among the public may be indicative of insufficient awareness and a general distrust of current clinical trial practices. A national survey study reported that only 25% of respondents were willing to participate in clinical trials (19), a markedly lower percentage compared to our findings. This discrepancy could potentially be attributed to variations in the demographic characteristics of the participants involved in the respective surveys.

The pressure of undergoing medical treatment emerged as a significant factor influencing respondents’ willingness to participate the DCTs. Participants tended to exercise caution in selecting treatment methods when they felt they had some control over their medical situation. However, when the pressure of medical treatment exceeded a certain threshold, individuals became more inclined to explore new treatment options. Consequently, it is essential to tailor communication approaches to individuals based on their level of pressure to seek medical care. For those experiencing mild pressure to seek medical care, recruiters should employ strategies aimed at alleviating their concerns through psychological counseling, thereby encouraging voluntary participation in clinical trials. Conversely, individuals facing high levels of pressure to seek medical care require a more nuanced approach. Before deciding whether to participate in clinical trials, it is crucial to emphasize the potential advantages and disadvantages of such involvement. This approach ensures that individuals fully understand the implications of their decision within the context of their medical circumstances.

Moreover, medical expenditure demonstrated a significant correlation with both participants’ awareness rates and their willingness to participate in DCTs. Individuals with high medical expenditures often undergo frequent medical treatments for various illnesses. Consequently, they tend to possess a better understanding of hospital procedures and have more opportunities to encounter ongoing DCTs, thereby increasing their awareness of drug clinical trial-related knowledge and their willingness to participate. It’s worth noting that DCTs typically offer drugs and relevant examinations free of charge, thereby reducing the overall treatment costs for patients. Consequently, individuals with lower incomes may exhibit a higher willingness to participate in DCTs compared to those with higher incomes. This is because participation in such trials presents an opportunity to access treatment at no cost, potentially alleviating the financial burden associated with medical care.

Some limitations existed in the survey. This study employed convenience sampling and snowball sampling techniques, resulting in a restricted representativeness and broad diversity in terms of occupation and age within the sampled population, inducing a measure of bias in the research outcomes. WeChat groups were established in each community for questionnaire distribution and retrieval, facilitated by the use of two-dimensional code for online data collection. For this reason, there are significant limitations regarding external validity, as the sample population may not be representative of the entire Chinese population. Moreover, calculating the survey response rate is unfeasible, and assessing internal validity becomes challenging.

The variables “pressure to seek medical care” and “financial pressure” are moderately subjective and liable to bias. These variables were incorporated to evaluate the impact of “pressure to seek medical care” and “financial pressure” on clinical trial participation. Patients encountering heightened “pressure to seek medical care” and “financial pressure” manifest a higher inclination to partake in clinical trials and are also more prone to reap benefits from their participation.

The limited sample size of this study may not fully represent the broader situation in China. Therefore, future research should consider employing hierarchical randomization methods for demographic factors, expanding the sample size, and incorporating more representative questions related to DCTs.

In summary, our findings provide valuable insights into the public’s awareness of DCTs. Strengthening effective publicity efforts regarding drug clinical trials-related knowledge in the future is crucial. It is evident that the public’s willingness to participate in clinical trials in China is relatively low and significantly influenced by their professional backgrounds and relevant experiences. Therefore, it is imperative to conduct scientific outreach initiatives tailored to the public’s needs. This approach will not only enhance public awareness but also foster a greater willingness to participate in clinical trials. Ultimately, such efforts will contribute to the smooth progression of DCTs, promote advancements in medical care, and serve to improve public health overall.

## Data availability statement

The raw data supporting the conclusions of this article will be made available by the authors, without undue reservation.

## Ethics statement

Ethical review and approval was not required for the study of human participants in accordance with the local legislation and institutional requirements. Written informed consent from the patients/participants was not required to participate in this study in accordance with the national legislation and the institutional requirements.

## Author contributions

LW: Writing – review & editing, Supervision. RZ: Writing – original draft. JL: Data curation, Formal analysis, Writing – review & editing. RX: Writing – original draft. LZ: Writing – original draft. EL: Writing – original draft. YZ: Writing – original draft, Data curation.

## Footnotes

^1^ https://www.nmpa.gov.cn/ylqx/ylqxjgdt/20150818200801163.html
