# Supplementary material for: An online survey on public awareness of drug clinical trials in inland cities of northern China
Source: Front Public Health. 2024 Apr 11;12:1276536. doi: 10.3389/fpubh.2024.1276536 (PMC11043599; doi:10.3389/fpubh.2024.1276536)
Supplement: Supplementary file 1 [file Data_Sheet_1.docx]

**Online Survey Questionnaire on Public Awareness of Drug Clinical Trials**

Dear friend,

Have you heard of 'Drug Clinical Trials '? Have you ever participated in one before? In order to better understand public awareness and attitudes towards clinical trials, and to ensure the smooth progress of these trials, as well as promote overall advancements in medical practices, we have launched this research survey. We kindly ask for your voluntary participation in this survey, and to anonymously complete the questionnaire. Your honest and earnest responses are crucial in ensuring the authenticity and reliability of this research. Please note that the survey results will not be used for commercial purposes. We truly appreciate your willingness to take a few minutes of your time to participate in this survey. Thank you for your support.

**Section I: General Information**

**1. Your age:**

18-20 years old

21-30 years old

31-40 years old

41-60 years old

above 60 years old

**2. Gender:**

Male

Female

**3. Your educational attainment:**

Under junior college

Junior college

Undergraduate

Postgraduate

**4. Your occupation:**

Administrative organization

Public institution

Enterprise

Student

Farmer

Retired

Other

**5. Work fields:**

Medical-related

Non-medical related

**6. Household type:**

Urban

Rural

**7. Marital status:**

Unmarried

Married

**8. City type:**

First-tier city

Second-tier city

Third-tier city

Fourth-tier city and below

**9. Current income:**

0-2000 RMB

2001-3000 RMB

3001-5000 RMB

5001-8000 RMB

above 8000 RMB

**10. Medical insurance:**

Public medical insurance

Urban employee medical insurance

Urban and rural resident medical insurance

Out-of-pocket medical expenses

Private/commercial insurance

Other

**11. Medical expenditure:**

Negligible

Less than 50% of monthly income

Equal to or greater than 50% of monthly income

**12. Pressure to seek medical care:**

None

Mild

Moderate

Severe

**13. Financial pressure:**

None

Mild

Moderate

Severe

**Section II: Cognitive Survey:**

**1. Are you aware of or have you heard about clinical trials for medications?**

□ Yes □ No □ Not familiar

**2. Clinical trials for new medications are a necessary and crucial step in drug development.**

□ Yes □ No □ Not familiar

**3. Clinical trials for new medications are divided into phases I, II, III, and IV.**

□ Yes □ No □ Not familiar

**4. Generally, Phase I clinical trials involve healthy volunteers as participants.**

□ Yes □ No □ Not familiar

**5. Participants are assigned to a treatment group through random allocation, rather than based on personal or physician preferences.**

□ Yes □ No □ Not familiar

**6. During the trial, participants might be assigned to a placebo group, an experimental drug group, or a control group.**

□ Yes □ No □ Not familiar

**7. During the trial, it is possible that neither the participants nor the doctors are aware of which group the participants are assigned to (drug group or control group).**

□ Yes □ No □ Not familiar

**8. Clinical trials can only be conducted when the potential benefits outweigh the potential risks of the research.**

□ Yes □ No □ Not familiar

**9. Participants may not directly benefit from participating in clinical trials.**

□ Yes □ No □ Not familiar

**10. Clinical trial medications should be provided free of charge and should not involve any fees.**

□ Yes □ No □ Not familiar

**11. Recruitment advertisements for participants should not include inducive information such as compensation amounts or treatment effects.**

□ Yes □ No □ Not familiar

**12. Participants have the right to withdraw from the clinical trial at any time.**

□ Yes □ No □ Not familiar

**13. Participation in clinical trials is voluntary, and no one can be forced to participate.**

□ Yes □ No □ Not familiar

**14. Participants must sign an informed consent form before participating in clinical trials.**

□ Yes □ No □ Not familiar.

**15. The informed consent form should fully inform participants of the potential benefits and risks of the trial.**

□ Yes □ No □ Not familiar.

**16. During clinical trials, participants' privacy and personal information should be adequately protected.**

□ Yes □ No □ Not familiar.

**17. Not all adverse events that occur during clinical trials will necessarily lead to compensation.**

□ Yes □ No □ Not familiar.

**18. Commercial insurance should be purchased for trial participants during clinical trials.**

□ Yes □ No □ Not familiar.

**19. Ethics committees can safeguard participants' rights during clinical trials.**

□ Yes □ No □ Not familiar.

**20. Ethics committee opinions and decisions are not influenced by any other factors.**

□ Yes □ No □ Not familiar

**Section III: Willingness to Participate:**

**1. Have you ever participated in a drug clinical trial?**

Yes

No

**2. Are you willing to participate in a drug clinical trial?**

Willing

Unwilling

**3. Are you willing to recommend friends, relatives, etc., to participate in clinical trials?**

Willing

Unwilling

**4. Motivation for willingness to participate in drug clinical trials.**

Economic reasons

Access to more convenient medical care

Acceptance of new treatment methods

Promotion of medical progress

Trust in doctors

**5. Reasons for Refusing to Participate in Drug Clinical Trials.**

Insufficient understanding of drug clinical trials

Concerns about trial risks

Concerns about personal privacy and information leakage

Unwillingness to be treated as a "Test Subject"

Unwillingness to spend more time and effort

**6. Are you willing to obtain knowledge and information related to drug clinical trials?**

Willing

Unwilling

**7. Ways in which you wish to obtain knowledge and information related to drug clinical trials.**

Promotional posters

Science popularization videos and articles

Multimedia platforms such as Tiktok or WeChat or the internet

Introduction by medical professionals

Other

**8. Are you willing to become a volunteer clinical trial promoter?**

Willing

Unwilling

**9. Pathways through which you have learned about clinical trials.**

Attending physicians

Acquaintances or patients

Social media platforms such as WeChat or MicroBlog

Pharmaceutical companies

Other

Never heard of it
